# AMPK–mTOR Signaling and Cellular Adaptations in Hypoxia

**DOI:** 10.3390/ijms22189765

**Published:** 2021-09-09

**Authors:** Yoomi Chun, Joungmok Kim

**Affiliations:** Department of Oral Biochemistry and Molecular Biology, School of Dentistry, Kyung Hee University, Seoul 02447, Korea; ychun@khu.ac.kr

**Keywords:** hypoxia, AMPK, mTORC1, hypoxia-inducible factor (HIF), hypoxic cellular adaptations

## Abstract

Cellular energy is primarily provided by the oxidative degradation of nutrients coupled with mitochondrial respiration, in which oxygen participates in the mitochondrial electron transport chain to enable electron flow through the chain complex (I–IV), leading to ATP production. Therefore, oxygen supply is an indispensable chapter in intracellular bioenergetics. In mammals, oxygen is delivered by the bloodstream. Accordingly, the decrease in cellular oxygen level (hypoxia) is accompanied by nutrient starvation, thereby integrating hypoxic signaling and nutrient signaling at the cellular level. Importantly, hypoxia profoundly affects cellular metabolism and many relevant physiological reactions induce cellular adaptations of hypoxia-inducible gene expression, metabolism, reactive oxygen species, and autophagy. Here, we introduce the current knowledge of hypoxia signaling with two-well known cellular energy and nutrient sensing pathways, AMP-activated protein kinase (AMPK) and mechanistic target of rapamycin complex 1 (mTORC1). Additionally, the molecular crosstalk between hypoxic signaling and AMPK/mTOR pathways in various hypoxic cellular adaptions is discussed.

Oxygen (O_2_) is a key factor for driving cellular metabolism in mitochondria to maintain cellular energy homeostasis for cell proliferation and growth. Therefore, under low oxygen conditions that do not reach the cellular requirement (hypoxia, 0.5–2% oxygen), vertebrates should reprogram the metabolic pathways and the corresponding regulatory circuits in response to the stressful conditions caused by hypoxia, thereby promoting cell survival [1,2]. Considering oxygen is delivered by the bloodstream, it should be noted that hypoxia is accompanied with nutrient starvation in many physiological settings in mammals. Therefore, it is necessary to consider the crosstalk between hypoxic signaling and nutrient signaling and the consequent metabolic changes. In the nutrient signaling, AMP-activated protein kinase (AMPK) and mechanistic target of rapamycin complex 1 (mTORC1) play an important role in balancing cellular energy homeostasis by sensing cellular ATP and nutrient (glucose and amino acids) levels. They are also key upstream regulators for triggering autophagy, an essential cellular homeostasis program that removes harmful and damaged cellular materials and provides cellular energy sources and building blocks. The AMPK–mTOR pathway cooperates with autophagy to fine-tune metabolic activity in response to stressful conditions. In this review, we will introduce and discuss the current understanding of AMPK–mTOR signaling and cellular adaptations in hypoxia.

## 1. AMPK as a Cellular Energy Gauge

**Figure 1:**
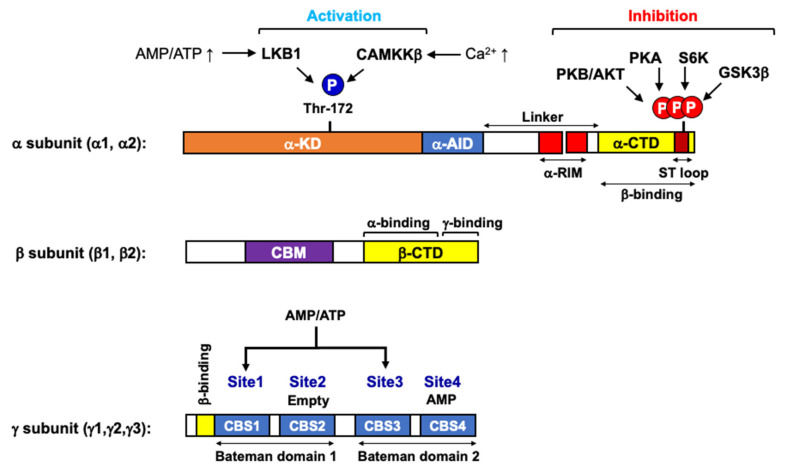
Functional domains of AMPK subunits. AMPKα catalytic subunit (α1 and α2) has an N-terminal kinase domain (α-KD) containing Thr-172 for activation by upstream kinases (LKB1 and CAMKKβ), autoinhibitory domain (α-AID), and two regulatory-subunit interacting motifs (α-RIM), and a C-terminal domain binding to the β-subunit (α-CTD). The ST-loop within α-CTD can be highly phosphorylated by PKB/AKT, PKA, S6K, and GSK3β, which leads to the inactivation of AMPK. The AMPKβ scaffold subunit (β1 and β2) has a carbohydrate-binding module (CBM, a target region for direct AMPK activators, such as A-769662 and salicylate), a C-terminal domain (β-CTD) containing α-subunit binding site, and a domain for γ-subunit interaction. The AMPKγ direct energy-sensing subunit (γ1, γ2, and γ3) has four cystathionine-β-synthase domains (CBS1–4), which form two Bateman domains that create four adenosine nucleotide-binding sites (Site1–4). Site2 always appears to be empty and Site4 has a tightly bound AMP, whereas Site1 and Site3 represent the regulatory sites that bind AMP, ADP, or ATP competitively.

AMPK structure for directly sensing cellular ATP level. AMPK is primarily regulated by cellular energy level [3,4]. As its name indicates, AMP activates AMPK. AMP is a by-product of cellular adenylate kinase reaction, an ATP buffer system catalyzing the conversion of two ADPs into one ATP and one AMP at close to equilibrium. Therefore, the AMP/ATP ratio, as the square of the ADP/ATP ratio, varies [5]. This means that changes in the cellular AMP level are a more sensitive indicator of cellular energy status than changes in ADP or ATP levels [6]. Once activated in ATP-depleting conditions, AMPK acts to restore energy homeostasis by activating catabolic pathways, including glucose uptake, glycolysis, and fatty acid oxidation, which facilitates ATP generation. Simultaneously, AMPK inhibits ATP-consuming anabolic pathways such as fatty acid synthesis, gluconeogenesis, and protein synthesis. AMPK is a heterotrimeric protein kinase complex consisting of a catalytic subunit (α1, α2) and two regulatory subunits (β1, β2 and γ1, γ2, γ3), of which the γ-subunit functions as an energy sensor by directly binding to adenosine nucleotides, ATP, ADP, or AMP (Figure 1) [3,6]. It contains four tandem cystathionine β-synthase (CBS1–4) motifs [7]. A single tandem pair of CBS modules forms a ‘Bateman domain’ to provide two adenosine nucleotide-binding sites [8], therefore, there are four potential adenine nucleotide-binding sites (Site1–4) in AMPK. X-ray crystallographic analyses have shown that Site1, Site3, and Site4, but not Site2, are capable of binding to ATP, ADP, or AMP in a competitive manner [7,9]. Mutagenesis further indicates that Site3 and Site4 are important for AMPK allosteric activation, and it has been proposed that Site3 primarily contributes to the allosteric activation of AMPK by AMP. AMP binding to Site3 causes conformational changes in the AMPK complex by rearranging the regulatory subunit interacting motif on the α-subunit (α-RIM) in close proximity to AMP bound Site3 on γ-subunit [10]. It causes the release of an intramolecular autoinhibitory domain (α-AID) from a kinase domain (α-KD) on the α-subunit, allowing AMPK to adopt an active conformation.

Regulation of AMPK activity by phosphorylation. In addition to allosteric regulation, phosphorylation on multiple regions of the α-subunit plays an important role in the regulation of AMPK activity (Figure 1) [3]. First, the phosphorylation of Thr172 within the activation loop on α-KD is a key determinant for AMPK activation. It is believed that the conformational changes upon AMP binding not only makes the AMPK complex more sensitive to Thr172 phosphorylation on α-subunit by upstream kinases but also protects this site from dephosphorylation by protein phosphatase PP2A and PP2C [11,12]. Coordination of AMP binding and AMPKα Thr172 phosphorylation causes synergistic activation (>1000-fold increase in AMPK activity), conferring the high sensitivity of AMPK in response to small changes in cellular energy status. Three different kinases have been identified as upstream kinases phosphorylating AMPKα at Thr172. First, the tumor suppressor LKB1 (liver kinase B1) is a primary AMPK kinase, especially in response to cellular energy stress [13,14,15]. Interestingly, recent studies have shown that Axin, originally discovered as a component of Wnt signaling whose deficiency leads to the duplication of the body axis [16], forms a stable complex with LKB1, and AMP allows AMP-bound AMPK complex to bind to the Axin-LKB1 complex, thus promoting Thr172 phosphorylation [17]. Moreover, it is has been shown that the Axin-LKB1 complex is translocated into lysosomes in response to ATP depletion, in which case AMPK is activated [18]. This finding provides an important clue to resolve the AMPK–mTOR–autophagy molecular network triad in the lysosome, where the amino acid sensitive mTOR complex I and nutrient-recycling autophagy program are activated. Second, Ca^2+^/calmodulin-dependent protein kinase 2 (CaMKKβ) phosphorylates Thr172 of AMPKα in response to the elevated intracellular Ca^2+^ concentration, independently of any change in cellular AMP/ATP ratio [19,20]. Lastly, Thr172 can be phosphorylated by TGF-β-activated protein kinase (TAK1), but the physiological conditions under which TAK1 phosphorylates AMPK remain unclear [21]. Accumulating reports have demonstrated that the phosphorylation of the serine/threonine-rich loop (ST-loop) on AMPKα also plays a role in the regulation of AMPK, mostly by inhibition [3,22]. The kinases corresponding to this inhibitory phosphorylation include cyclic AMP-dependent protein kinase (PKA), PKB/Akt, and p70 S6 kinase 1 (S6K1). PKA has been reported to inhibit AMPK during gluconeogenic periods by directly phosphorylating AMPKα1 at Ser485 (equivalent to Ser491 in AMPKα2) [23]. PKB/Akt also phosphorylates the same site to inhibit AMPK, which was proposed as a mechanism for the inactivation of AMPK by insulin [24]. Similarly, S6K1 inhibits AMPK by phosphorylating AMPKα2 at Ser491, accounting for how leptin inhibits AMPK in the hypothalamus [25]. Glycogen synthesis kinase 3 (GSK3) [26], protein kinase D1 (PDK1) [27], and protein kinase C (PKC) [28] are also reported to phosphorylate various residues in the ST-loop and inhibit AMPK. Although the underlying mechanism remains to be uncovered, these inhibitory phosphorylations on the ST-loop may represent a negative regulatory circuit to turn off AMPK signaling when the proliferative metabolic signaling is forced to work, for example, in cancers harboring a constitutively active PKB/Akt mutation.

Metabolic regulation by AMPK. The impact of AMPK on the metabolism is largely observed in the metabolic pathways for glucose and fatty acids, two main cellular energy sources [29]. AMPK promotes glucose uptake. AMPK phosphorylates TBC domain family member 1 (TBC1D1) and thioredoxin-interacting protein (TXNIP), which collectively induces the translocation of glucose transporters (GLUT1 and GLUT4) onto the plasma membrane [30]. AMPK activates glycolysis by phosphorylating 6-phosphofructo-2-kinase/fructose-2,6-biphosphatase (PFKFB) while inhibiting glycogenesis (glycogen synthesis) by suppressing glycogen synthase (GYS) [29]. AMPK also controls overall cellular lipid metabolism through direct phosphorylation and concomitant inactivation of acetyl-CoA carboxylase 1 (ACC1), a rate-limiting enzyme in fatty acid synthesis producing malonyl-CoA from acetyl-CoA. In contrast to ACC1, ACC2 has a mitochondrial targeting sequence in its amino terminus. It makes the inhibition of ACC2 by AMPK an important mechanism that can accelerate fatty acid oxidation by relieving the inhibition of carnitine palmitoyltransferase 1 (CPT1) by malonyl-CoA on the mitochondria outer membrane [3,22]. Furthermore, AMPK phosphorylates and inhibits 3-hydroxy-3-methyl-glutaryl-coA reductase (HMGCR), a key enzyme in the mevalonate pathway that produces cholesterol and other isoprenoids. AMPK also promotes lipid absorption and release by phosphorylating lipases such as hormone-sensitive lipase (HSL) and adipocyte-triglyceride lipase (ATGL) [31,32]. In addition, AMPK can regulate metabolism at the transcriptional level. AMPK inhibits the transcriptional induction of gluconeogenesis via phosphorylation and nuclear exclusion of cyclic AMP-regulated transcriptional coactivator 2 (CRTC2) and Class II histone deacetylases (HDACs), which are necessary for the transcription of gluconeogenic genes [33,34]. Similarly, AMPK phosphorylates and inhibits sterol regulatory element binding protein 1 (SREBP1), a master transcription factor for lipogenic enzymes [35]. Phosphorylation of nuclear factor-4α (HNF4α) and carbohydrate-responsive element binding protein (ChREBP) by AMPK was also proposed to regulate the transcription of key glycolytic and lipogenic enzymes [36,37]. Interestingly, AMPK was reported to increase mitochondrial biogenesis via the peroxisome proliferator-activated receptor-γ coactivator 1α (PGC-1α), in which AMPK may directly activate PGC-1α by phosphorylation [38] or indirectly by activating an NAD^+^-dependent protein deacetylase Sirtuin-1 (SIRT1), which deacetylates and activates PGC-1α [39]. These metabolic reprogramming activities of AMPK are also closely related to the cell cycle progression. Interestingly, AMPK was reported to be directly involved in cell cycle regulation. It has been extensively reported that the activation of AMPK with AMP-mimetic 5-aminoimidazole-4-carboxamide ribonucleoside (AICAR) causes cell cycle arrest in various cell types in vitro and in vivo [40,41,42]. Although it is not clear whether AMPK directly phosphorylates and stabilizes p53, these studies indicate that the activation of AMPK accumulates p53, followed by an increase in p21/CIP, a G1 cell cycle inhibitor. Moreover, a report has demonstrated that AMPK activity is required for proper mitotic progression and cytokinesis, in which the AMPK (especially AMPKα2)-meditated phosphorylation of protein phosphatase 1 regulatory subunit 12C (PPP1R12C) plays an important role [43]. Consistent with AMPK, its upstream kinase LKB1 was also shown to be associated with p53 [44] and induce p21/CIP expression for cell cycle arrest in a p53-dependent manner in G361 melanoma cells [45].

## 2. mTOR Complex and Its Amino Acid Sensing Modules

**Figure 2:**
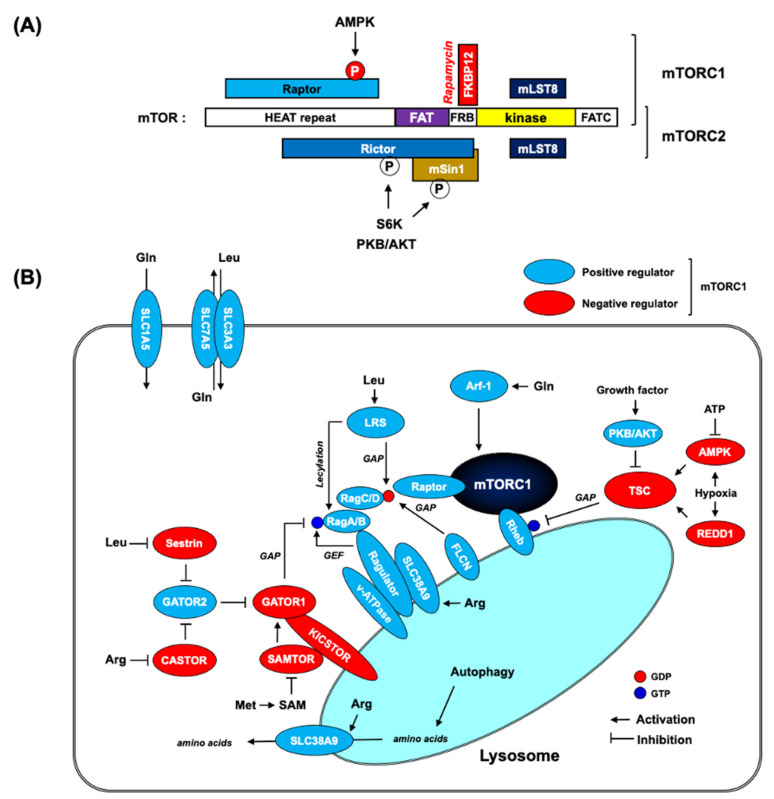
mTORC and its nutrient signaling modules. (**A**) A schematic representation of mTOR domain structure and mTORC subunits. mTOR is composed of a huntingtin-elongation factor 3-regulatory subunit A of PP2A-TOR1 (HEAT) repeat, a FRAP-ATM-TRRAP (FAT) domain, a FKBP12-rapamycin binding (FRB) domain, a catalytic domain (kinase), and a FAT domain at the C terminus (FATC). mTORC1 includes mTOR, regulatory-associated protein of mammalian target of rapamycin (Raptor), and mammalian lethal with sec-13 protein 8 (mLST8). Raptor is a phosphorylation target of AMPK, which leads to mTORC1 inactivation upon AMPK activation. mTORC2 contains mTOR, rapamycin-insensitive companion of mTOR (Rictor), mammalian stress-activated MAP kinase-interacting protein 1 (mSin1), and mLST8. The binding of Rictor and mSin1 masks the FRB domain on mTOR to prevent FKBP12–rapamycin binding, thereby rendering mTORC2 insensitive to rapamycin. mTORC2 is shown to be phosphorylated by PKB/AKT, as well as a downstream kinase S6K in mTORC1 signaling, but it is debated whether their physiological significance entails activation or inhibition. (**B**) mTORC1 and amino acid signaling network. mTORC1 is activated by Rheb on lysosomes. Therefore, localization of mTORC1 is required for activation, which is dependent on the intracellular amino acid level. Rag GTPase is a major arm for transmitting information on intracellular amino acid into mTORC1. In an amino acid-rich condition, the active Rag GTPase (GTP-loaded RagA/B and GDP-loaded RagC/D) binds to Raptor to recruit mTORC1 onto lysosomes. Many different amino acid-sensing molecules directly or indirectly regulate GTP/GDP loading status on Rag GTPase complex. Additionally, Rheb is negatively regulated by the TSC complex, a GAP for Rheb. The TSC complex integrates many different inputs (growth factors, cellular energy level, and oxygen level) to fine-tune Rheb for the tight regulation of mTORC1 signaling in response to various extracellular and intracellular cues. The proteins shown in the figure are not drawn to scale.

Two mTOR complexes. mTOR complex (mTORC) coordinates cell growth and metabolism by integrating growth factor signaling at the nutrient (mostly amino acids) level [46,47]. mTORC exists in two distinct protein kinase complexes (mTORC1 and mTORC2, Figure 2a). mTORC1 is a master regulator of protein synthesis by direct phosphorylation to activate p70 ribosomal protein S6 kinase1 (S6K1) and inactivate eukaryotic translation initiation factor 4E-binding protein 1 (4E-BP1). Additionally, mTORC1 triggers a cellular homeostatic degradation program, autophagy, by regulating autophagy-initiating protein kinase ULK1 complex. mTORC2 is a key upstream molecule that activates the phosphoinositide 3-kinase (PI3K)-PKB/AKT pathway by directly phosphorylating PKB/AKT at Ser473 on its well-conserved hydrophobic motif. mTORC1 and mTORC2 are defined by their unique subunits, Raptor and Rictor, respectively. Importantly, mTORC1 is sensitive to rapamycin, but mTORC2 is resistant to acute rapamycin treatment. Recent three-dimensional structural analyses have demonstrated that mTORC2-specific Rictor blocks the FKBP12–rapamycin complex binding site on mTOR, thereby making mTORC2 insensitive to rapamycin [48,49]. Nonetheless, a long-term rapamycin treatment can inhibit mTORC2 signaling by disassembling the complex [50,51]. There were also reports showing that micromolar concentrations of rapamycin inhibit both mTORC1 and mTORC2, while the lower concentrations of rapamycin in the nanomolar range only target mTORC1 [52]. The physiological significance of mTORC1 and mTORC2 signaling in cancer biology may explain the underlying mechanism behind the higher dose of rapamycin that is needed for mTORC1 inhibition in anti-cancer treatment in clinic trials [53]. Interestingly, phosphatidic acid (PA), a central metabolite of membrane lipid biosynthesis, has been reported to participate in mTORC1 activation. PA is a hydrolytic product of phosphatidylcholine (PC) by phospholipase D (PLD). The inhibition of PA by either pharmacological (1-butanol) or genetic (PLD knockdown by RNAi) approaches results in a decrease in mTORC1 signaling, reducing the phosphorylation of both S6K1 and 4E-BP1 [54,55]. However, PA does not seem to directly stimulate mTORC1 activity [56]. Instead, it may function to relieve the inhibitory input of rapamycin to mTORC1. PA has been demonstrated to interact with the FKBP12–rapamycin complex binding site of mTOR (FRB domain), thereby competing with the FKBP12–rapamycin complex for mTOR binding. In fact, the increase in cellular PA levels renders cells less sensitive to rapamycin [57]. However, it should be noted that RNAi screening in flies showed that *Drosophila* PLD (dPLD) knockdown did not cause any phenotype change in dTOR-dependent cell growth [58]. Moreover, biochemical analysis revealed that although the FRB domain is well-conserved in dTOR in flies, the critical Arg2109 residue in the mTOR FRB domain for PA binding is not conserved in dTOR [54].

mTORC1 and AMPK. Functionally, mTORC1 interacts with AMPK at the level of tuberous sclerosis complex (TSC) and Raptor. TSC is a heterotrimeric complex composed of TSC1, TSC2, and TBC1D7. It acts as a GTPase-activating protein (GAP) for lysosomal Rheb [59], which directly binds to and activates mTORC1, although the molecular details are still unclear [60]. AMPK phosphorylates TSC2 to increase GAP activity toward Rheb, thereby inhibiting mTORC1. AMPK also directly phosphorylates mTORC1-specific Raptor, leading to 14-3-3 binding and the allosteric inhibition of mTORC1 [61]. A reciprocal regulation of AMPK and mTORC1 by PLD and its metabolite, PA, has also been demonstrated [62]. It has been shown that the inhibition of PLD stimulates AMPK signaling to increase AMPKα Thr172 phosphorylation and its downstream target ACC1 phosphorylation, while PA was reported to decrease AMPK signaling in an mTORC1-dependent manner. Interestingly, this study also revealed that AMPK negatively regulated PLD1 activity. Considering that Rheb binds to and stimulates PLD activity in a GTP-dependent manner [56], the suppression of AMPK in response to elevated PLD activity may provide a positive feedback loop by inhibiting TSC GAP activity, thereby leading to the activation of both PLD and mTORC1 by GTP-bound Rheb. Elevated PLD activity is frequently observed in many cancers [63], therefore, this regulatory feedback circuit in the PLD–AMPK–mTORC1 axis may reinforce tumor growth and proliferative signal by inhibiting AMPK and simultaneously activating mTORC1. Consistent with this notion, there have been many efforts to use AMPK activators and mTOR inhibitors as anti-cancer drugs (Table 1). Furthermore, there is a report showing that the AMPK activator, AICAR, can enhance the efficacy of rapamycin in human cancer cells [64].

mTORC1 and nutrient sensing modules. Limitation of amino acid supply quickly turns off mTORC1 signaling. mTORC1 pathway includes many different amino acid sensors for the activation on lysosome (Figure 2b) [47,76]. Rag GTPase complex, consisting of two different Rag GTPases (RagA/B and RagC/D), plays an essential role in connecting mTORC1 with amino acid sensing [77,78]. Biochemical studies demonstrate that GTP-loaded RagA/B in complex with GDP-loaded RagC/D is necessary for mTORC1 activation and this active Rag GTPase complex preferentially binds to Raptor in mTORC1 [77]. Experiments in mice harboring a constitutively active allele of RagA have shown that the resulting active Rag GTPase complex kept mTORC1 active, even in nutrient starvation condition [79]. Unlike the Rheb GTPase, Rag GTPases do not directly activate mTORC1 in vitro, but it is required for lysosomal localization and activation of mTORC1 in response to amino acid stimulation [77,80]. These studies showed that mTORC1 anchoring on lysosomes is no longer sensitive to amino acid starvation while under the control of growth factor and Rheb. Interestingly, Rag GTPases do not have any lipid-targeting motifs, but the Rag GTPase complex is able to be localized on lysosomes through the pentameric Ragulator complex, consisting of p18, p14, MP1, HBXIP, and C7orf59 (also known as LAMPTOR1–5) [80,81]. Notably, these studies showed that Ragulator acts as a guanine nucleotide exchange factor (GEF) for RagA/B, thereby, activating mTORC1 in the presence of amino acids. Many studies have identified a number of amino acid sensors that integrate the information about cytosolic and lysosomal amino acid concentrations with mTORC1 signaling [47,76]. Of the nutrient sensing complexes that transmit cytosolic amino acid signals to the Rag GTPase complex, GATOR1 complex, a heterotrimeric complex consisting of DEPDC5, NPRL2, and NPRL3, directly regulates the activity of the Rag GTPase complex [82]. It is a GTPase-activating protein (GAP) toward the Rag GTPase complex, hydrolyzing GTP-bound RagA/B and inhibiting mTORC1 signaling. GATOR1 is localized on lysosomes by the KICSTOR complex (which contains KPTN, ITFG2, C12orf66, and SZT2), which is required for the nutrient-mediated control of mTORC1 [83,84]. In addition, GATOR1 interacts with GATOR2, a pentameric complex of WDR59, WDR24, MIOS, SEH1L, and SEC13, which inhibits GATOR1 to activate mTORC1 [82]. The following studies have demonstrated that GATOR2 functions as a signaling platform to regulate mTORC1 by interacting with various amino acid sensors. Upon leucine starvation, the cytosolic leucine sensor Sestrin2 binds and inhibits GATOR2, preventing lysosomal recruitment of mTORC1 [85,86]. Similarly, in the absence of arginine, a cytosolic arginine sensor CASTOR also binds to and inhibits GATOR2. Arginine binding to CASTOR disrupts CASTOR–GATOR2 interaction, thereby activating mTORC1 [87,88]. In the case of arginine, there is another arginine sensor, SLC38A9. It monitors amino acid levels inside the lysosomal lumen and defines the lysosomal branch of the nutrient sensing machinery [89,90]. SLC38A9 functions as an arginine-gated pump to transport lysosomal amino acids into the cytosol, such as leucine, leading to mTORC1 activation [91]. This efflux activity may represent a mechanism by which the amino acids from the autophagic degradation inside lysosomes can activate mTORC1 signaling after prolonged starvation. In parallel, the lysosomal v-ATPase has also been reported to interact with the Rag–Ragulator complex to regulate the nucleotide-loading state of the Rag–GTPase complex [92]. It was shown that an increase in the lysosomal amino acid concentration induces a conformational change in v-ATPase to decrease its interaction with the Ragulator complex. Interestingly, the GEF activity of the Ragulator complex toward RagA/B has been shown to be regulated by v-ATPase [81]. In contrast to GATOR1, a GAP for RagA/B, the folliculin (FLCN)–folliculin interacting protein2 (FNIP2) complex is shown to act as a GAP for RagC/D, which activates the mTORC1 pathway in the presence of amino acids [93,94]. Finally, an S-adenosylmethionine (SAM) sensor, SAMTOR, inhibits mTORC1 by binding to GATOR1 and KICSTOR under methionine or SAM deprivation [95]. This finding suggests that mTORC1 can respond not only to amino acids but also to their metabolic products. In parallel with the Rag–GTPase complex axis, there are additional amino acid sensors for mTORC1. First, yeast and mammalian studies have demonstrated that glutamine can stimulate mTORC1 independently of Rag GTPase complex [96,97]. These reports have shown that glutamine could promote mTORC1 translocation to the lysosome without functional Rag GTPase complex (RagA/B knockout), but it was still dependent on the lysosomal v-ATPase. Instead, it requires ADP ribosylation factor 1 (Arf-1) GTPase, a key regulator in vesicle trafficking. In addition, the cooperation of two glutamine transporters on the plasma membrane, SLC1A5 and SLC7A5/SLC3A3, is reported to function in glutamine-dependent mTORC1 activation [98]. SLC1A5 increases intracellular glutamine by an influx of extracellular glutamine, which drives a bidirectional amino acid transporter, SLC7A5/SLC3A3, to move intracellular glutamine out and extracellular essential amino acids, such as leucine, in to activate mTORC1. However, glutamine was also reported to activate mTORC1 through a Rag GTPase complex-dependent mechanism [99]. Additionally, leucyl-tRNA synthetase (LARS) is reported as an intracellular leucine sensor of mTORC1 via both Rag GTPase-dependent and -independent mechanisms. LARS has been shown to directly interact with Rag GTPase complex and activate mTORC1 by functioning as a GAP for RagD [100]. Recently, LARS has also been reported to mediate leucylation on Lys 142 of RagA to activate mTORC1 [101].

Metabolic regulation by mTORC1. mTORC1 signaling plays a fundamental role in various biosynthetic pathways [47,102]. mTORC1 drives lipid synthesis by activating two key lipogenic transcription factors, sterol regulatory element binding protein (SREBP) and peroxisome proliferator-activated receptor-γ (PPARγ). mTORC1 indirectly activates SREBP by phosphorylating lipin 1, a phosphatidic acid phosphatase [103]. Lipin 1 promotes nuclear remodeling and blocks the translocation of SREBP into the nucleus as well as its transcriptional activity. Once lipin 1 is phosphorylated and inactivated in an mTORC1-dependent manner, SREBP becomes active to initiate lipogenic programs. In fact, mice with adipose-specific loss of mTORC1 have been shown to have smaller and fewer adipocytes and be resistant to high fat diet induced obesity [104]. Additionally, the downstream effector of mTORC1, S6K1, has been reported to regulate the commitment of embryonic stem cell to adipogenic progenitors by regulating the adipogenic program [105]. Although the underlying mechanism is still largely unknown, mTORC1 signaling participates in the gene expression for lipid homeostasis in a manner dependent on the nuclear receptor PPARγ [106]. mTORC1 also plays an essential role in one-carbon metabolism for nucleotide biosynthesis. mTORC1 induces a mitochondrial methylenetetrahydrofolate dehydrogenase 2 (MTHFD2) in tetrahydrofolate (THF) cycle for de novo purine synthesis by activating the transcription factor ATF4 [107]. Additionally, S6K1 has been shown to phosphorylate and activate a carbamoyl phosphate synthetase 2-aspartate transcarbamoylase-dihydroorotase (CAD), a rate-limiting enzyme in pyrimidine biosynthesis [108,109]. mTORC1 can also feature in glucose metabolism, especially in response to hypoxia and related physiological conditions, such as cancers. mTORC1 upregulates the transcription factor hypoxia inducible factor 1α (HIF1α) to enhance the expression of glycolytic enzymes, which favors glycolysis over oxidative phosphorylation [110,111]. mTORC1-dependent SREBP activation also increases metabolic flux through the pentose phosphate pathway, providing NADPH (a reducing power for lipid synthesis as well as ROS scavenging system) and ribose-5-phosphate (a precursor for nucleotide synthesis) [110]. Moreover, mTORC1 has been shown to be involved in ketogenesis in hepatocytes [112]. Mice with liver-specific loss of TSC1, which leads to a constitutively active mTORC1 signaling, did not produce ketone bodies on fasting. mTORC1 impairs the activity of PPARα, a master transcriptional regulator of ketogenic genes, by promoting the nuclear accumulation of nuclear receptor corepressor 1 (NcoR1). Interestingly, mTORC1 was also shown to stimulate mitochondria biogenesis by promoting the formation of the yin yang 1 (YY1)-PPARγ coactivator 1α (PGC1α) active transcriptional complex [113]. This appears to be in line with reports demonstrating that mTORC1 enhances the translation of nuclear- encoded mitochondrial transcripts through its downstream effector, 4E-BP1, to increase the capacity of ATP synthesis for cell growth [114].

## 3. Cellular Adaptions to Hypoxia

**Figure 3:**
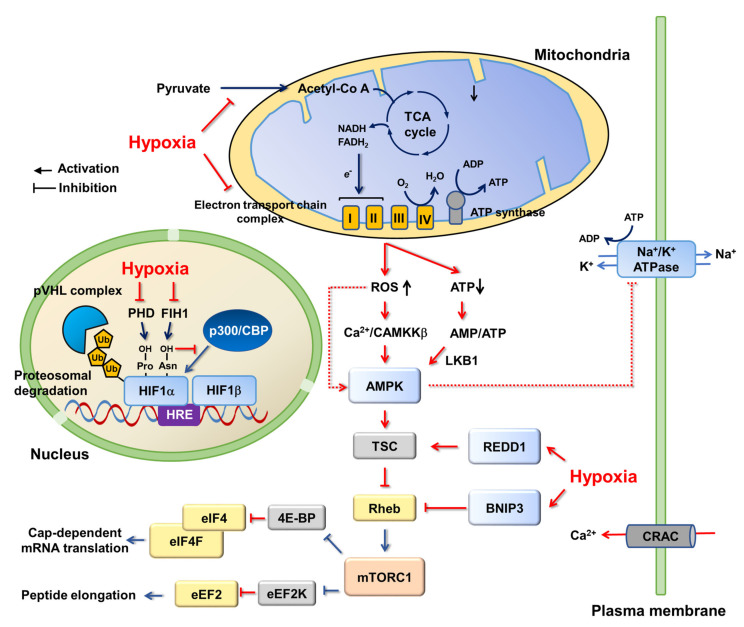
AMPK, mTORC1, and HIF1 in hypoxia. Hypoxia inhibits the mitochondrial electron transport chain complex to impair ATP synthesis. It can cause increases in intracellular ROS level, which increases intracellular Ca^2+^ to activate AMPK, independent of any AMP/ATP change. When hypoxia lasts hours, intracellular AMP/ATP level is increased, which further enhances or maintains AMPK activation. AMPK inhibits mTORC1 via TSC, which is also directly activated by hypoxia through REDD1. In addition, hypoxia-induced BNIP3 disrupts Rheb-mTORC1 interaction, thereby leading to mTORC1 inhibition. Inactivation of mTORC1 in hypoxia causes the deceleration of protein translation at both initiation (by inhibiting eIF4F mRNA cap-binding complex) and elongation (by activating eEF2 kinase, a negative regulator of elongation factor eEF2) steps. Additionally, hypoxia stabilizes a heterodimeric transcription factor HIF by inhibiting HIFα proline hydroxylase PHD, which prevents the interaction between HIFα and E3 ubiquitin ligase pVHL complex. Molecular pathways regulated by hypoxia are shown in red.

AMPK regulation by hypoxia. Activation of AMPK under hypoxia has been reported in various tissues and cell types via different molecular mechanisms [115]. In general, AMPK is believed to be activated by accumulating AMP with respect to the decreasing ATP in hypoxic conditions (Figure 3). In this aspect, the LKB1-AMPK axis is highlighted as a main route for AMPK activation in hypoxia. The hypomorphic expression of LKB1 is reported to abrogate AMPK activation under hypoxia in smooth muscle cells, while a knockout of CaMKKβ had no effect on the activation of AMPK under hypoxia in mice [116]. Additionally, LKB1 is shown to be an essential upstream molecule for AMPK activation by hypoxia in lung epithelial cells [117]. Interestingly, hypoxia can increase intracellular Ca^2+^ and concomitantly activate CaMKKβ independently of any significant change in ATP and AMP level. Indeed, AMPK activation in HeLa and HEK293T cells under hypoxia was blunted by CaMKKβ inhibitor, STO-609 [118]. Additionally, in contrast to lung epithelial cells, CaMKKβ seems to be responsible for AMPK activation under hypoxia in alveolar epithelial cells [119]. Alternatively, although the underlying mechanism remains largely unknown, AMPK can be activated by reactive oxygen species (ROS) in this low oxygen condition [120]. ROS are produced in mitochondrial respiration (electron transport) for oxidative phosphorylation (OXPHOS) [121,122]. Even under normal conditions, it was estimated that ROS produced by mitochondria are about 1–2% of the total rate of oxygen consumption [123], therefore, cells have ROS scavenging enzymes such as superoxide dismutases (SODs), catalase (CAT), and glutathione peroxidases (GPX), as well as antioxidant agents, such as nicotinamide adenine dinucleotide phosphate (NADPH) and glutathione (GSH) [124]. Hypoxia is reported to increase cellular ROS levels mainly through targeting Complex III on the mitochondrial electron transport chain (ETC), accompanied by AMPK activation [125,126]. Accumulation of ROS by hypoxia damages mitochondria to blunt ATP synthesis and increase cellular AMP/ATP, which may represent a mechanism for AMPK activation [126]. In contrast, other reports have demonstrated that AMP concentration was not increased after hypoxia-induced ROS formation [125,127,128,129]. It was proposed that ROS can directly regulate AMPK activity. H_2_O_2_ was shown to induce oxidation and S-glutathionylation of cysteine residues (Cys299 and Cys304) on AMPKα in HEK293 and lung cells, resulting in the activation of AMPK [128]. However, there was an opposite result in cardiomyocytes, in which H_2_O_2_ and ischemia induced oxidation of other cysteine residues (Cys130 and Cys174) on α-subunit, which inhibited AMPK through aggregation of AMPK molecules and blockage of Thr172 phosphorylation by upstream kinases [130].

Hypoxia-inducible transcription factors, HIFs. The life obtaining biological energy from mitochondrial oxidative phosphorylation largely relies on oxygen for proliferation, growth, and survival [131]. Additionally, hypoxia arises in many different pathophysiological conditions, such as tumors. Once an initially avascular tumor has grown beyond the diffusion limits of oxygen, hypoxic microdomains develop. An important milestone in our understanding of cellular adaptions to hypoxia was the discovery of hypoxia inducible factors (HIF1 and HIF2): a heterodimeric transcription factor complex containing an oxygen-sensitive HIFα (present in hypoxia) and -insensitive HIFβ (constitutively expressed regardless of cellular oxygen level) (Figure 3) [132]. HIFs bind to hypoxia response elements in the promoter of many hypoxia-responsive genes, including those involved in cell survival, angiogenesis, glycolysis and invasion/metastasis. In normoxia, the HIF1α subunit is hydroxylated on two proline residues (Pro402 and Pro564) within the oxygen-dependent degradation (ODD) domain by prolyl hydroxylases (PHD), coupled with the oxidative decarboxylation of α-ketoglutarate (α-KG). The hydroxylated HIF1α is polyubiquitylated by the pVHL complex (pVHL-elongin B/elongin C-CUL2) and targeted for proteasomal degradation [133,134]. Hypoxia prevents the hydroxylation and consequent degradation of HIF1α subunits, leading to the formation of an active HIF1α-HIF1β transcription factor complex. The transcriptional activity of HIFs is further regulated by another member of the Fe^2+^ and α-ketoglutarate-dependent dioxygenase family, HIF asparaginyl hydroxylase or factor inhibiting HIF1 (FIH1) [133,134]. In normoxia, FIH1 inhibits HIFs by hydroxylating an asparagine residue within the C-terminal transactivating domain of the HIFα subunit to prevent recruitment of the transcription co-activators, p300/CBP [135]. Notably, PHD is under the control of metabolites in the tricarboxylic acid (TCA) cycle. Succinate and fumarate inhibit PHD by competing with its substrate α-ketoglutarate, causing an accumulation of the HIFα subunit, even in normoxia [136]. Additionally, hypoxia concomitant with acidosis causes the production of L-2-hydroxyglutarate (2-HG), a structural analog of α-KG, thereby suppressing PHD activity and increasing the protein level of HIFα subunit [137]. Interestingly, it was reported that AMPK was activated by treatment with the pan-hydroxylase inhibitor dimethyloxalylglycine (DMOG) in human colorectal adenocarcinoma cells, CaCo-2, and neonatal rat cardiomyocytes [138,139]. This activation appears to rely on Ca^2+^-CaMKKβ signaling, but not on LKB1 [138]. However, it is not clear whether the activation of AMPK by DMOG is mediated by PHD because a limited amount of functional proline-hydroxylation of non-HIF proteins by PHD has been documented [140,141,142] and, moreover, they are challenged by a recent in vitro PHD assay [143]. Considering the observation of asparagine hydroxylation in several non-HIF proteins, such as tankyrase [144], Notch-1 [145], and IκBα [146], FIH1 may be an alternative. Indeed, a possible link between AMPK and FIH1 was demonstrated in brown adipose tissue [147] and the human embryonic kidney cell, HEK293 [148]. Notably, there are some reports expecting crosstalk between AMPK and HIFs in hypoxia, although the molecular details are still largely unknown. It was reported that pharmacological or genetic inhibition of AMPK diminished HIF1 activation in hypoxia [149,150]. Additionally, it was proposed that AMPK may induce activating phosphorylation on HIF1α for its transcriptional activity [150,151]. However, there are conflicting reports showing upregulation of HIF1α under inactive AMPK in some cancer cells [152,153]. Similarly, AMPK has been shown to promote PHD activity [154]. This appears to be in line with the observation that AMPK increases α-ketoglutarate levels, an important cofactor for PHD [3].

mTORC1 and REDD1 in hypoxia. Although the activation of AMPK may provide a primary mechanism to inhibit mTORC1 in hypoxia, hypoxia can also directly regulate mTORC1 by a protein regulated in development and DNA damage response-1 (REDD1, also known as DDIT4) (Figure 3) [155,156]. REDD1 activates TSC complex by disrupting the inhibitory interaction between TSC2 and 14-3-3, thereby inactivating mTORC1. Hypoxia decreases the expression of microRNA-7 (miR-7), which binds to 3-UTR on REDD1 mRNA for degradation. Therefore, hypoxia relieves the repression of REDD1 expression by miR-7 [157]. In parallel, hypoxia induces mitochondrial protein Bcl-2/adenovirus e1B 19 kDa-interacting protein 3 (BNIP3) expression by HIF1, which can interfere with the interaction between mTORC1 and Rheb that inactivates downstream signaling [77,158]. In addition, hypoxia results in ataxia telangiectasia mutated (ATM)-dependent HIF1α phosphorylation, which appears to be required for REDD1 upregulation and mTORC1 downregulation [159].

Protein translation in hypoxia. Along with cellular adaptation at the transcriptional level via HIFs, down-regulation of energy consuming protein translation minimizes unnecessary ATP use and prevents the accumulation of unfolded (or misfolded) proteins by hypoxia, in which protein kinase R (PKR)-like endoplasmic reticulum (ER) kinase (PERK) and mTORC1 play an important role [131]. Accumulation of unfolded/misfolded proteins causes ER stress, followed by the unfolded protein response (UPR) [160]. As a UPR sensor, PERK phosphorylates eIF2α and inhibits translation initiation, relieving the proteotoxicity [161,162]. In parallel, inhibition of mTORC1 in hypoxia is responsible for turning off a cap-dependent mRNA translation by regulating the eIF4F complex. The eIF4F complex binds to 5′-cap of mRNA, which brings it to a ribosome. eIF4E is an essential component of eIF4F cap-binding complex, as well as a target of 4E-BP1, a direct substrate of mTORC1. mTORC1 phosphorylates 4E-BP1 to prevent 4E-BP1 binding to eIF4E, which allows for the formation of a functional eIF4F complex. Upon mTORC1 inactivation by hypoxia, 4E-BP1 inhibits eIF4F cap-binding complex assembly to decrease global protein translation [163]. mTORC1 also functions in the elongation step in the translation. Peptide elongation is mediated by eukaryotic elongation factors (eEFs), of which eEF2 is a target of hypoxic signaling [164,165]. In normoxia, mTORC1 phosphorylates eEF2 kinase, which is subject to proteasomal degradation. However, once mTORC1 is inactivated in hypoxia, the inhibitory phosphorylation on eEF2 kinase is decreased and the eEF2 kinase becomes stable to phosphorylate and inhibit eEF2 function [164]. In this translation-unfavorable condition, the hypoxia-responsive mRNA translation can occur by direct binding between ribosomes and the internal ribosome entry sites within the 5′-untranslated region (UTR) of the mRNAs, which allows the mRNAs to bypass eIF4F cap-binding complex dependent translation [166,167]. Notably, the translation of hypoxia-adaptive gene expression can be upregulated in hypoxia. Typically, the overexpression and activation of activating transcription factor 4 (ATF4) in hypoxia drives various genes’ expression, such as the genes involved in protein synthesis, antioxidant response, amino acid transport, metabolism, and autophagy, as a part of the integrated stress response [168,169,170]. Up-regulation of ATF4 translation in hypoxia is known to rely on eIF2α phosphorylation [168,171], which is resistant to mTORC1 inhibition. However, mTORC1 is also able to activate ATF4 signaling. It has been shown that mTORC1 induces ATF4-dependent expression of methylenetetrahydrofolate dehydrogenase 2 (MTHFD2), a key enzyme in the mitochondrial tetrahydrofolate (mTHF) cycle, to provide one-carbon units for de novo purine synthesis for cell growth and proliferation [107].

Mitochondrial respiration and ROS in hypoxia. In hypoxia, mitochondria are first in line to experience the change in oxygen level because the mitochondrial electron transport chain (ETC) is the largest single consumer of intracellular oxygen for the generation of ATP [131]. Although the underlying mechanism has yet to be fully elucidated, Complex I, II, and III are sensitive to hypoxia, but Complex IV appears to be rather resistant to it [131]. As a terminal ETC component, Complex IV (cytochrome c oxidase, COX) stops electron flows by delivering electrons to oxygen, producing two molecules of water in the process. It has a high affinity for oxygen (Km close to 0.1% oxygen), therefore, the ETC can function at near anoxic levels (around 0.5% oxygen) and cells can maintain minimum ATP levels to survive during hypoxia [172]. When hypoxia lasts for hours, in order to support basal metabolic demands, hypoxia switches the subunit in Complex IV in a HIF1-dependent manner by degrading COX4I1 and expressing COX4I2, which allows for the more efficient transfer of electrons to oxygen during hypoxia [173]. By contrast, hypoxia diminishes the activity of Complex I, II, and III [131]. Hypoxia induces microRNAs, including miR-210, to repress the expression of two important Fe-S cluster assembly factors (ISCU1 and ISCU2), thereby compromising Complexes I, II and III [174]. A benefit of reduced mitochondrial oxidative phosphorylation and ETC activity is to minimize mitochondrial ROS in response to acute and short hypoxia. In fact, many HIF1-dependent hypoxia responsive gene products diminish mitochondrial ROS, preventing cell death [173,175,176,177]. However, under long-term or severe hypoxic conditions, the overproduction of ROS results in severe damage on cells, leading to cell death. In response to acute or mild hypoxia, cells turn on the mechanisms to decrease cellular ATP demand by suppressing ATP-consuming processes, thereby decelerating oxygen consumption [178]. It has long been believed that the demand for cellular ATP is a major determinant of the cellular respiratory rate [179]. In fact, sodium/potassium pumps (Na^+^/K^+^ ATPase) account for 20–70% of the oxygen expenditure of mammalian cells [180]. This pump is rapidly inhibited by AMPK in hypoxia [127,181]. These studies have shown that hypoxia generates ROS to activate AMPK by CaMKKβ, but not by LKB1. Activated AMPK phosphorylates PKCζ, followed by a decrease in Na^+^/K^+^ ATPase on the plasma membrane via endocytosis in alveolar epithelial cells. In this condition, hypoxia increases cellular Ca^2+^ level by calcium release-activated calcium (CRAC) channels. CARC channels are responsible for store-operated calcium (SOC) entry, a major route of Ca^2+^ influx in nonexcitable cells, and are activated by the depletion of Ca^2+^ stores in ER [182]. The opening of CRAC channels refills the Ca^2+^ stores in ER, leading to long-lasting calcium signaling [183]. The ROS, in turn, causes the release of Ca^2+^ from the ER, and the Ca^2+^ influx through CRAC channels stimulates CaMKKβ, which is followed by AMPK activation.

**Figure 4:**
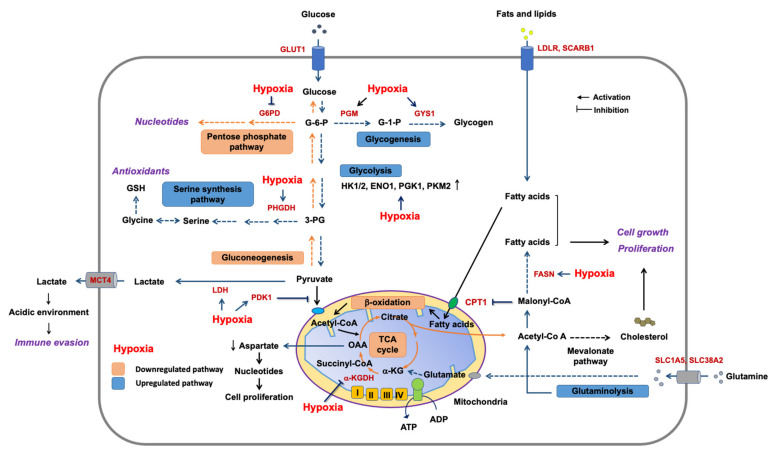
Metabolic adaptations to hypoxia. Glucose transporter1 (GLUT1), hexokinases1/2 (HK1 and HK2), enolase1 (ENO1), phosphoglycerate kinase1 (PGK1), pyruvate kinase (PKM2), pyruvate dehydrogenase kinase1 (PDK1), lactate dehydrogenase (LDHA), glutamine transporters (SLC1A5 and SLC38A2), α-ketoglutarate dehydrogenase (α-KGDH), fatty acid synthase (FASN), glucose-6-phosphate dehydrogenase (G6PD), phosphoglycerate dehydrogenase (PHGDH), phosphoglucomutase1 (PGM1), glycogen synthase1 (GYS1), LDL receptor (LDLR), and HDL receptor (SCARB1). Glucose-6-phosphate (G-6-P), glucose-1-phosphate (G-1-P), 3-phosphoglycerate (3-PG), α-ketoglutarate (α-KG), oxaloacetate (OAA), and glutathione (GSH).

Metabolic adaptation in hypoxia. Hypoxia profoundly influences many key metabolic pathways (Figure 4). Hypoxia decreases pyruvate influx into the TCA cycle in mitochondria by activating lactate dehydrogenase A (LDHA) and pyruvate dehydrogenase kinase 1 (PDK1) in a HIF1-dependent manner [176,184]. LDHA converts pyruvate to lactate. PDK1 phosphorylates and inhibits pyruvate dehydrogenase (PDH), an enzyme that produces acetyl-CoA from pyruvate to fuel the TCA cycle. The decrease in the metabolic flow into the TCA cycle eventually diminishes cellular aspartate level, which is largely provided by oxaloacetate from the TCA cycle [185]. Aspartate is necessary for nucleotide synthesis, suggesting that aspartate can be a limiting metabolite for tumor growth [131]. The hypoxia-induced metabolic reprogramming is one of the key features of tumors. An imbalance between vascular formation/organization and cell proliferation/growth in tumors results in both oxygen and nutrient starvation. It makes cancer cells rewire the metabolic pathways to ensure tumor progression in these unfavorable conditions. First, hypoxic area in tumors develops glucose uptake and the concomitant increase in glycolytic flux. HIF1 reprograms the metabolism by inducing the transcription of genes encoding glucose transporters (GLUT1 and GLUT3), hexokinases (HK1 and HK2), enolase (ENO1), phosphoglycerate kinase (PGK1), pyruvate kinase (PKM2), PDK1, and LDHA [127,184,186,187]. This metabolic reprogramming results in the accumulation of lactate and H^+^ in cytosol, which is secreted by monocarboxylic transporter (MCT4), sodium-hydrogen (Na^+^/H^+^) exchanger (NEH1), and carbonic anhydrase (CAR9) [131]. Extracellular lactate can then be taken up by other cancer cells or stromal cells, where it is used as a fuel for the TCA cycle [188,189]. Additionally, the lactate can be an alternative carbon source, replenishing the intermediates in the TCA cycle, especially in human non-small-cell lung cancers (NSCLC) [188]. Notably, the resulting lactate and H^+^ acidifies the tumor microenvironment to inhibit immune responses around cancers (tumor immune evasion) by suppressing the infiltrating T cells [190]. Hypoxia also upregulates glutamine uptake by increasing the gene expression of glutamine transporters (SLC1A5 and SLC38A2) [191,192]. Glutamine is a key anaplerotic substrate for the TCA cycle to fuel TCA intermediates, of which a citrate is converted into cytosolic acetyl-CoA to support lipid biosynthesis in hypoxia. In addition, hypoxia induces E3 ubiquitin-protein ligase SIAH1, which triggers ubiquitination and degradation of α-ketoglutarate dehydrogenase (α-KGDH, an enzyme catalyzing the oxidative conversion of α-ketoglutarate into succinyl-CoA in the TCA cycle). It blocks the flow of glutamine-derived α-ketoglutarate in the TCA cycle and promotes a reductive carboxylation of α-ketoglutarate to citrate for lipogenesis [191]. Additionally, hypoxia stimulates fatty acid synthase (FASN, a key enzyme in fatty acid synthesis) expression in an HIF1-dependent manner [186]. Hypoxic metabolic reprogramming also regulates ROS production [131]. Hypoxia decreases the expression of glucose-6-phosphate dehydrogenase (G6PD), thereby decreasing the flow into the pentose phosphate pathway [193]. However, at the same time, hypoxia increases phosphoglycerate dehydrogenase (PHGDH) expression to reinforce the serine biosynthesis for antioxidant responses, promoting stress resistance [193]. Additionally, glucose is redirected into glycogenesis under hypoxia by overexpression of phosphoglucomutase 1 (PGM1) and glycogen synthase 1 (GYS1). Building glucose stores in preparation for glucose deprivation may constitute an auxiliary mechanism [187].

Autophagy and hypoxia. In hypoxia, cells also activate autophagy for survival. Autophagy (i.e., macroautophagy) is a catabolic program to remove harmful cellular contents, such as damaged organelles and protein aggregates, from lysosomes. Simultaneously, it provides the energy and new building blocks required to promote cell survival in stressful environments [194,195]. Hypoxia is often accompanied by nutrient depletion, which activates AMPK and simultaneously inactivates mTORC1, triggering an autophagy program. AMPK and mTORC1 cooperatively regulate autophagy at the level of two autophagy-initiating kinase complexes, ULK1 and PIK3C3/VPS34 [196,197]. mTORC1 phosphorylates ULK1, which interferes with ULK-AMPK interaction. Once AMPK becomes active in hypoxia conditions, the inhibitory phosphorylation of ULK1 by mTORC1 is undone, and, in turn, AMPK phosphorylates and activates ULK1 to initiate autophagy [196]. Additionally, AMPK phosphorylates Beclin 1, a component of PIK3C3/VPS34 complex, to activate ATG14L (or UVRAG)-containing pro-autophagy PIK3C3/VPS34 complex, which triggers autophagy [197]. Similarly, mTORC1 phosphorylates UVRAG to inhibit the complex by recruiting the inhibitor protein RUBICON into the UVRAG-associated complex. Upon mTORC1 inactivation, this inhibitory UVRAG phosphorylation is diminished to release UVRAG from RUBICON, allowing the UVRAG–HOPS complex to interact with a lysosome to trigger autophagosome maturation [198]. Additionally, hypoxia-induced BNIP3 and BNIP3-like (BNIP3L/Nix) can induce autophagy, especially mitophagy (a selective degradation of mitochondria by autophagy) [199]. BNIP3 and BINP3L/Nix function as adaptors connecting damaged mitochondria to autophagosomes (a double-membrane structured autophagic vesicle that delivers the destructive cargo into lysosomes) via their LC3 (an essential autophagosome marker on autophagosome)-interacting region (LIR) [200]. Although the corresponding kinase is unknown, multiple phosphorylations on BNIP3/BNIP3L appear to be decisive for the function of those receptors and for mitophagy induction [201]. In parallel, BNIP3/BNIP3L also triggers autophagy by regulating Beclin 1 [202]. It was demonstrated that Beclin 1 forms a complex with Bcl-2 (or Bcl-xL) to inhibit autophagy under normoxic conditions. In hypoxia, BNIP3/BNIP3L binds to Bcl-2, liberating Beclin 1 from the complex to leave a functional PIK3C3/VPS34 complex. The resulting BNIP3/BNIP3L-Bcl-2/Bcl-xL complex functions to prevent cell death from hypoxia. Additionally, there are reports showing that BNIP3 can trigger the translocation of Drp1 into mitochondria, resulting in mitochondrial fragmentation and mitophagy induction in cardiomyocytes [203]. Importantly, Drp1 localization into mitochondria and subsequent mitochondrial fission seem to be a prerequisite for BNIP3-mediated mitophagy in cardiomyocytes.

Hypoxia is an important pathophysiological condition that can induce massive cellular adaptive responses. It is generally accompanied by nutrient starvation, therefore, hypoxic signaling must be closely linked to nutrient signaling. Indeed, accumulating reports have shed light on the communication of two important signaling pathways, AMPK and mTORC1, with hypoxic signaling. Their crosstalk can be found in a variety of cellular adaptations (mitochondria respiration, ROS production, protein translation, metabolic reprogramming, and autophagy) to hypoxia. These mechanisms are integral inputs for fine-tuning responses to hypoxic stress.

## Figures and Tables

**Table 1:** Trials using AMPK activators and mTOR inhibitors as anti-cancer drugs.

**AMPK Inhibitors**		**Status**	**Study Model**	**Reference**
AICAR	ZMP, AMP mimetic	Preclinical study	Cancer cell lines (prostate, glioblastoma, colon)	[65,66,67]
A769662	a direct activator	Preclinical study	Cancer cell lines (breast, melanoma, lung)	[68]
MT63-78	a direct activator	Preclinical study	Cancer cell lines (prostate), animal model	[69]
OSU-53	allosteric activator	Preclinical study	Cancer cell lines (thyroid), animal model	[70]
Metformin	inhibits mitochondrial electron transport chain complex I	Clinical trials (phase II)	Breast, prostate, pancreatic cancers	[71]
**mTOR Inhibitors**		**Status**	**Study Model**	**Reference**
Rapamycin		Preclinical	Animal model (Pancreatic cancer)	[72]
Everolimus Temsirolimus	a derivative of rapamycin	US FDA approved	Renal cell carcinoma (RCC)	
ICSN3250	targets PA binding to FRB domain on mTOR	Preclinical study	Cancer cell lines (colon)	[73]
LY3023414	a competitive ATP-binding inhibitor	Clinical trials (phase I)	Solid tumor or lymphoma	[74]
AZD8055	a competitive ATP-binding inhibitor	Preclinical study	Animal model (ovarian clear cell carcinoma)	[75]

## Data Availability

Not applicable.
